# Supplementary material for: Electrically Driven Reprogrammable Vanadium Dioxide Metasurface Using Binary Control for Broadband Beam Steering
Source: ACS Appl Mater Interfaces. 2022 Sep 1;14(36):41186–95. doi: 10.1021/acsami.2c10194 (PMC9478939; doi:10.1021/acsami.2c10194)
Supplement: Supplementary file 1 — am2c10194_si_001.pdf [file am2c10194_si_001.pdf]

Supplementary Information

Electrically driven reprogrammable vanadium dioxide metasurface using binary control for broadband beam-steering

Matthieu Proffit[1], Sara Pelivani[1], Pascal Landais[2] and A. Louise Bradley [1,3*]

[1]School of Physics and AMBER, Trinity College Dublin, D02PN40, Dublin 2, Ireland
[2]School of Electronic Engineering, Dublin City University, Glasnevin, Dublin 9, Ireland
[3]IPIC, Tyndall National Institute, T12 R5CP, Cork, Ireland

Corresponding author email : bradlel@tcd.ie

CONTENTS

I.	Phase profile calculation	2
II.	Non-normal incidence	2
III.	2D case	3
IV.	Peak power	4
V.	Number of resolvable points	6
VI.	Thermal design	8
VII.	Stability Analysis	11
VIII.	Inverse design	12
IX.	Individual antenna wiring	14

I. Phase profile calculation

We calculate the phase profile in order to maximise amplitude at a given angle in the Fraunhofer conditions. As we use coherent incident light, all incident rays are in phase. In order to have maximum amplitude at $\theta = \theta_r$ requires $\Delta\Phi_i + \Delta\Phi_s + \Delta\Phi_r = 0[2\pi]$, with:

- $\Delta\Phi_i = -k_0.\Delta x.sin(\theta_i)$ the incident phase delay
- $\Delta\Phi_s$ the phase difference at the metasurface level
- $\Delta\Phi_r = -k_0.\Delta x.sin(\theta_r)$ the reflected phase delay

We can calculate $\Delta\Phi$ any angle θ (with $\Delta\Phi = k_0.\Delta x.sin(\theta)$), but only for the angle of anomalous reflection θ_r do we verify $\Delta\Phi_i + \Delta\Phi_s + \Delta\Phi_r = 0[2\pi]$.
The following equation is obtained :

$$sin(\theta_r) = \frac{\Delta\Phi_s}{\Delta x}\frac{\lambda}{2\pi} - sin(\theta_i) \quad (1)$$

In this section, and the following, we use the following sign conversion in order to maintain the x-y plots consistent with a increase in angle from left to right.

- + : clockwise angle
- - : counterclockwise angle

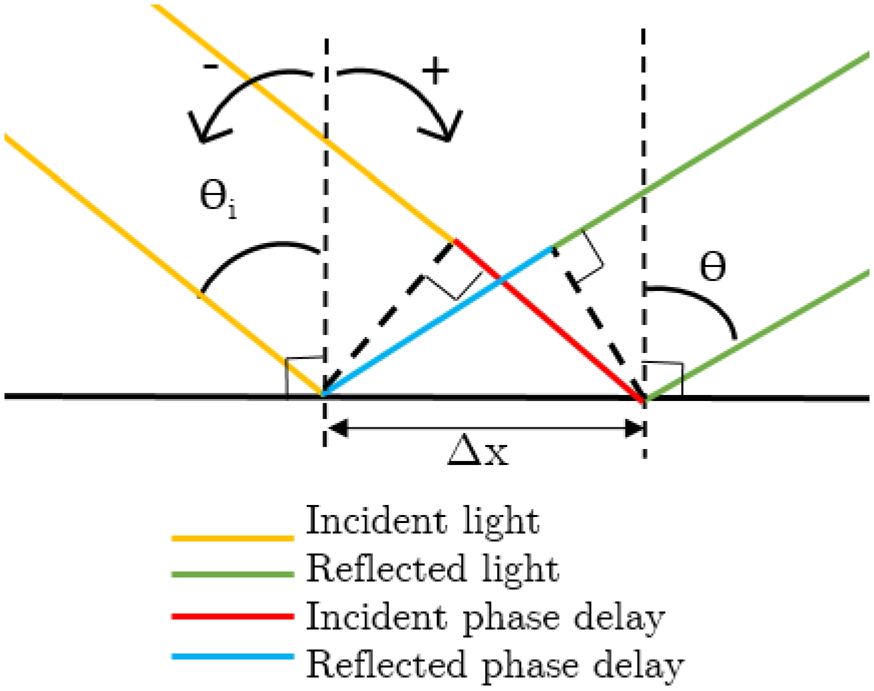

Figure S1: Phase delay schematic

II. Non-normal incidence

As stated in the main paper, we can break the symmetry in the far-field pattern by introducing an off-normal angle of incidence, pushing the $-\theta_r$ peak out of the region of interest or suppressing it (if $|sin(\theta_r)| > 1$). This region is defined by a single angle θ_{max} as to keep it symmetric around the array normal vector as $[-\theta_{max}; \theta_{max}]$.

In the following derivation, we assume a negative angle of incidence (in the second quadrant) as a sign convention to maintain $\theta_{max} > 0$.

From equation 1, we define α the steering parameter that can be tuned between $\pm\lambda/2p$ where p is the system's periodicity. This maximum value is achieved when we are in a "grating" configuration : the switching pattern is ON-OFF-ON-OFF-ON...

$$\alpha = \frac{\Delta\Phi\lambda}{2\pi\Delta x} = sin(\theta_r) + sin(\theta_i) \quad (2)$$

In the case of binary control, the array state is going to be in the same state with $\alpha = \pm\alpha_0$ as can be seen in Figure 2-b in the main paper. We therefore restrict ourselves to using positive values of α. We note that with $\alpha = 0$, the metasurface is a simple mirror with $\theta_r = -\theta_i$. This corresponds to one of our boundaries and correspondingly :

$$\theta_{max} = -\theta_i \quad (3)$$

Similarly, we want to reach θ_{max} with a maximum phase gradient, which corresponds to $\alpha = \lambda/2p$. We re-arrange equation 2 and obtain :

$$sin(\theta_{max}) = \frac{\lambda}{2p} + sin(\theta_i) \quad (4)$$

Substituting equation 3 in equation 4 , we find the following :

$$\theta_{max} = -\theta_i = arcsin(\frac{\lambda}{4p}) \quad (5)$$

We show in equation 5 that decreasing periodicity to values lower than than $\lambda/4$ does not increase the region of interest of the array, however it may decrease the discretisation effects near the boundary for extreme angles. However as we will avoid grazing incidence in practice, we can choose a spatial period longer than $\lambda/4$. For example, if we choose $\theta_{max} = 60°$, we have $p = \lambda/4.arcsin(\pi/3) = 0.289\lambda$, which is $p = 447$ nm at $\lambda = 1550$ nm.
Similarly, by choosing a periodicity $p = \lambda/3$, we have $\theta_i = arcsin(\frac{3}{4}) = 48.6°$.
We can also note that if we add π phase successively at each antenna ($\Delta\Phi/\Delta x = \pi/p$, we obtain a different phase profile that also corresponds to a beam steering configuration. The steering parameter can be rewritten as $\alpha = \alpha_0 + \lambda/2p$, there will be constructive interference corresponding to α_0 in that case (in the region of interest) : this justifies the upper bound on α.

III. 2D CASE

We stated in the main paper that the 2D case equations could be drawn easily from the 1D case, in this section we derive them and show how binary control can be applied to 2D beam steering. This analysis is purely theoretical and does not apply to the electrically activated VO_2 structure presented in the paper. The practical implementation of a 2D phased array would not be feasible without significant changes. Several 2D phased arrays have however been proposed and the theory drawn in this section could be directly applied to control these devices [1, 2].
In order to simplify the analysis, we do not directly use spherical coordinates but instead use θ and γ the angles between the chosen direction and the x-axis and the y-axis,respectively, as shown in Figure S III.. It is possible to go from a spherical coordinate system [pol, az] to this θ,γ system using the following variable change :

$$sin(\theta) = sin(pol).cos(az) \quad sin(\gamma) = sin(pol).sin(az) \quad (6)$$

Using this variable choice, the Fraunhofer conditions allow us to completely separate both variables and consider the phase shift associated with θ and γ separately. For the mth antenna located at a position $[x_m, y_m]$, we have two phase shifts associated with θ and γ defined as :

$$\Phi_x = x_m.k_0.(sin(\theta_i) + sin(\theta_r)) \quad (7)$$

$$\Phi_y = y_m.k_0.(sin(\gamma_i) + sin(\gamma_r) \quad (8)$$

Two antennas located at the same x (respectively y) position in the array will have the same phase constraint Φ_x (respectively Φ_y). For a rectangular array, this makes the calculations slightly easier.

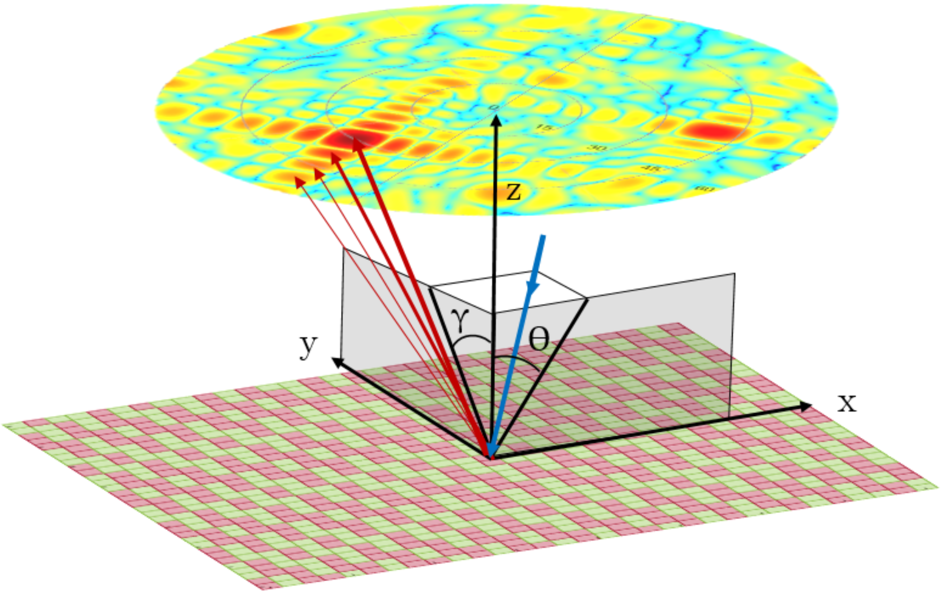

Figure S2: 2D Beam steering schematic - choice of angle system. The incident beam is in blue, the reflected field from the array in red (line width proportional to the intensity of the field in the arrow direction) and the coordinate system in black. We show the definition of γ and θ vis-à-vis with the incident beam

The phase selection algorithm is the same as in 1D binary control but we have to consider the sum of the two constraints. If $\Phi = \Phi_x + \Phi_y \ [2\pi]$ is in $[-\pi/2, \pi/2[$, then the antenna is left in its OFF state, similarly, if Φ is in $[\pi/2, 3\pi/2[$ we turn the antenna in its ON state.

The array state derived from this algorithm is more visual than its 1D counterpart as can be seen in Figure S3, the resulting far-field is very satisfactory demonstrating beam-steering in 2 dimensions as shown in Figure S4. These plots were obtained for an array with x-periodicity and y-periodicity of $\lambda/3$, angle of incidence θ_i and γ_i of $45°$ and $45°$ and desired angle of anomalous reflection θ_r and γ_r of $-10°$ and $30°$.

Direct simulations are significantly more time consuming for 2D beam steering as we have to sum $N_\theta * N_\gamma$ antennas over a far-field discretised in N^2 points. Which means the complexity of the algorithm is increase to the power two, it is still a reasonable computational time even for commercial desktops, provided that the far-field is not discretised too finely.
The difference with the ideal case (with perfect continuous 2π phase shift) is shown in Figure S5, we use a logarithmic scale to better visualise small amplitudes.

IV. Peak power

The far-field amplitude profile produced by a phased array in the Fraunhofer conditions is calculated from equation 9.

$$A(\theta)^{1/2} = E(\theta) = \sum_{m=1}^{N} E_m(\theta) e^{j.(\omega.t + \Phi_m + k_0 x_m \sin(\theta_i) + k_0 x_m \sin(\theta))} \tag{9}$$

Where $A(\theta)$ is the energy distribution with respect to angle θ, θ_i is the incident angle, Φ_m and x_m are the tunable phase shift applied at reflection by the antenna and the position of the mth antenna in the array. Regarding $E_m(\theta)$, we can apply the usual cosine distribution : $E_m(\theta) = E_m \cos(\theta)$. By integrating equation 1, we get the phase profile along the array length in equation 10:

$$\Phi(x)_{steering} = \Phi_0 + \frac{2\pi(\sin(\theta_r) + \sin(\theta_i))}{\lambda} x \tag{10}$$

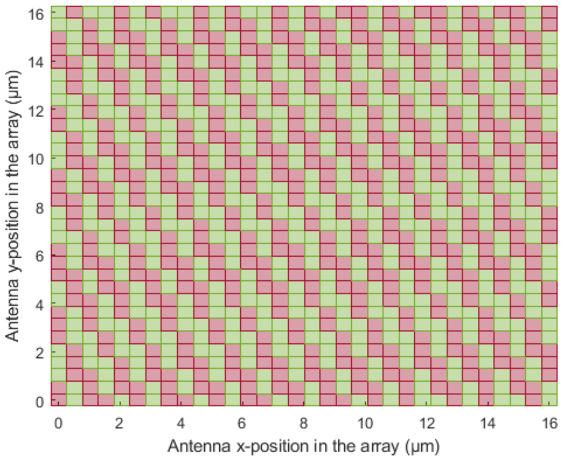

Figure S3: Array state in 2D

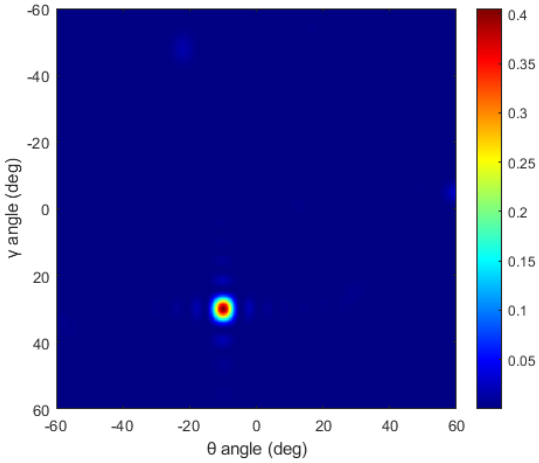

Figure S4: Far-field in 2D

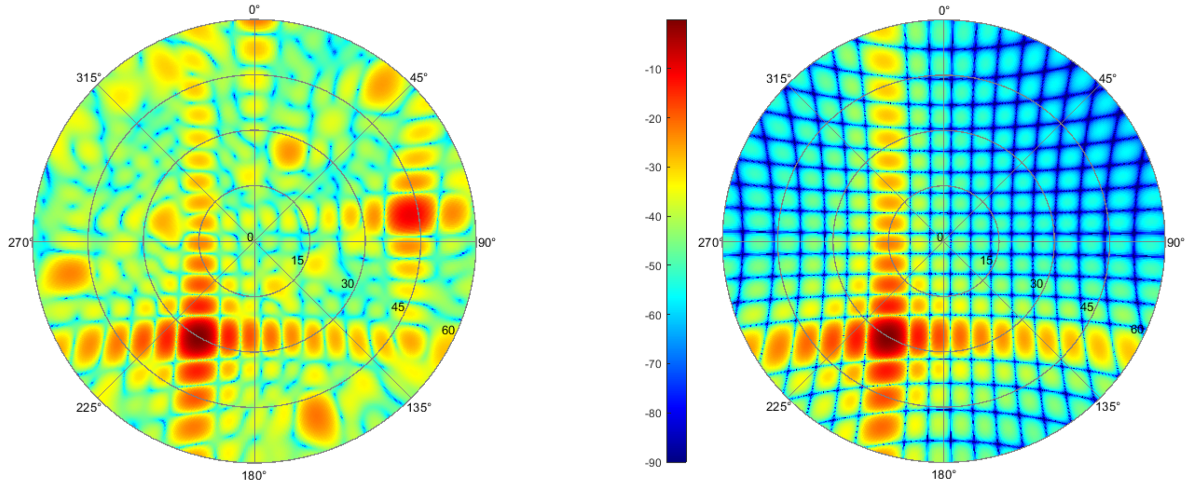

Figure S5: 2D Binary control compared to ideal control - far-field amplitude in logarithmic scale, azimuthal angle 210°, polar angle 30°

In this ideal case, we calculate the peak far-field amplitude at $\theta = \theta_r$ (where all the antennas interfere constructively in the far-field) with equation: 11.

$$A(\theta_r)^{1/2} = E(\theta_r) = \sum_{m=1}^{N} E_m(\theta_r) e^{j\omega t} = E_m(\theta_r) \sum_{m=1}^{N} e^{j\omega t} = N.E_m(\theta_r).e^{j\omega t} \quad (11)$$

However, in binary control, there is a discrepancy between the ideal phase profile and the actual reflected phase, $\Delta\Phi_m$ for each antenna given by equation 12.

$$\Delta\Phi_m = \Phi_m + k_0 x_m \sin(\theta_i) + k_0 x \sin(\theta) \quad (12)$$

The antenna state selection algorithm minimises $\Delta\Phi_m$ but given the coarse 1-bit discretisation of this control mechanism, we are left with a phase error randomly distributed in $[-\pi/2; \pi/2]$ in the general case (when the anomalous reflection angle does not correspond to an exact "grating profile" or periodic arrangement). The peak power at $\theta = \theta_r$ in this case is calculated in equation 13.

$$A_d(\theta_r)^{1/2} = E_d(\theta_r) = \sum_{m=1}^{N} E_m(\theta_0) e^{j(\omega t + \Delta\Phi_m)} \simeq N E_m(\theta_r) e^{j\omega t} \frac{1}{\pi} \int_{-\pi/2}^{\pi/2} e^{j\Delta\Phi} d\Delta\Phi = \frac{2N E_m(\theta_r) e^{j\omega t}}{\pi} \quad (13)$$

We can compare it to the ideal case in equation 14 and see that there is a power loss of about 60% due to destructive interference in the main beam compared to the ideal case. These destructive interference are solely due to the coarse discretisation in binary control and not to any inaccuracy in its implementation. This control mode is therefore not ideal in applications where high power efficiency is needed.

$$\frac{A_d(\theta_r)}{A(\theta_r)} = \frac{|2N E_m(\theta_r)|^2}{|\pi N E_m(\theta_r)|^2} = \frac{4}{\pi^2} \simeq 0.4053 \quad (14)$$

Note that this derivation is applicable to the 1D case as well as the 2D case as it does not assume anything about the arrangement of the antennas. The only assumption was that the discretisation error is randomly distributed in $[-\pi/2; \pi/2]$, which remains true in the general case for 2D arrays. In the 2D case, the summation from m=1 to N should be understood as N being the total number of antennas regardless of their position.

V. Number of resolvable points

As stated in the paper, the number of arrangements that correspond to a beam steering configuration scales with N^2, we calculated this number by brute-force simulations over a range of N (up to 256) to confirm that claim but calculating the exact number of arrangements remains an open mathematics problem.
The number of possible configuration for an array comprised of N antennas is 2^N but the vast majority of these arrangements do not correspond to beam steering and they would produce a random far-field interference pattern. In order to calculate the number of "legal configurations" that correspond to a constant phase gradient (with discretisation errors of course), we use the following brute-force algorithm :

- Choose a very small step-size, typically $0.01°$ and boundaries that correspond to the region of interest. Initialise a configuration list to null.
- Conduct a sweep with these parameters for all angles in this region separated by the step-size, calculate the array arrangement
- Compare the array configuration with the latest addition in the configuration list : if the arrangement is not new, discard it and go to the next iteration. If it is, add it to a memory list for future comparison.
- If in doubt, double check if a smaller angular step-size leads to an increased number of beam steering configurations found

$N_{antenna}$	N_{config}
2	2
4	6
8	24
16	89
32	378
64	1536
128	6207
256	24897

Table S 1: Number of array configurations corresponding to beam steering versus number of antennas

This algorithm is not optimised for efficiency but we can calculate the number of beam steering configurations in a few seconds for up to 256 antennas which is fine in practice. Table 1 and Figure S6 summarise the results of this algorithm. An excellent fit with a 2nd degree polynomial $N_{config} = 0.38 * N^2$ is found, the derivation of the exact value of the polynomial constant is beyond the scope of this paper however it is not surprising to see N_{config} scaling with N^2 exactly.
Since the beam produced by a phased array has a given angular width, it is possible to distinguish two beams at different angles of anomalous reflection only if they are sufficiently angularly separated. The FWHM is usually defined as the angular width of the beam, two beams can be resolved it they are separated by more than their FWHM. The FWHM is inversely proportional to the number of antennas, and therefore the number of resolvable points in the far field is directly proportional to N. Therefore, we will quickly have more beam-steering configurations than resolvable points, for reasonable values of N like $N = 32$, we already have 378 individual beam-steering arrangements, while the number of resolvable points is around 20. Hence the beam steering can be considered effectively continuous even if only a discrete number of points are obtained. The jumps between one angle to the next is imperceptible given the beam width, even for rather low N values.

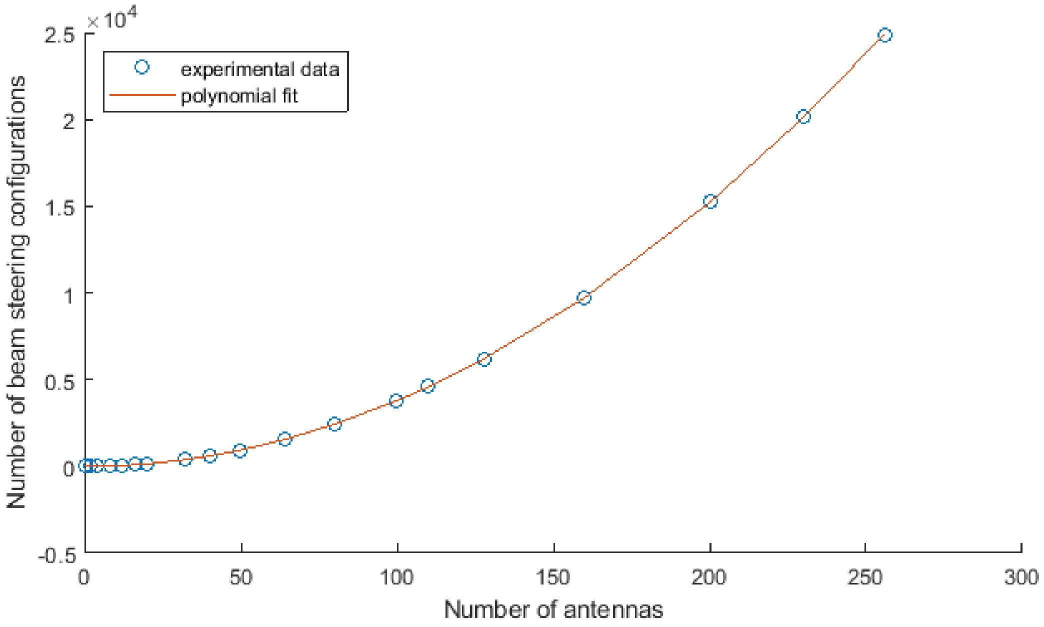

Figure S6: Number of beam-steering array arrangements versus number of antennas

VI. Thermal design

A. Array heat generation

We average out the heat generation at the array scale per unit surface, and this value is then used in the IHS model. In order to do so, we consider the energy consumed in a square of dimensions $p * p$ where p is the periodicity, this corresponds to a length of p for the single antenna. We are rearranging equation 5 from the main paper to calculate Q_{vol} over a volume $V = p * w * t_{Au-thermal}$.

$$E_d = \frac{V * Q_{vol}}{S} = \frac{t_{Au-thermal} * p * w * Q_{vol}}{p^2} = \frac{t_{Au-thermal} * p * w * \Delta T * k}{t_{SiO_2} * t_{Au-thermal} * p^2} = \frac{w * \Delta T * k}{p * t_{SiO_2}} \quad (15)$$

If we use $p = 517$ nm, $w = 245$ nm, $\Delta T = 25°$, $k = 1.4$ W/m/K and $t_{SiO_2} = 600$ nm, we calculate with equation 15 : $E_d = 2770\ W/cm^2$ (realistically, only half of this value over the array surface as half the antennas are ON at a given time on average). Note that we can calculate the energy consumption in each antenna, we assume an antenna length of N*p in order to have a square array:

$$E_{antenna} = Q_{vol} * t_{Au-thermal} * w * N * p \quad (16)$$

With the same parameters as above and N=64, we have $E_{antenna} = 0.47mW$, this number is remarkable compared to external phase shifters where power consumption is several times higher (1.7mW/π [3], 20mW/π [4]), it drops to $E_{antenna} = 0.24mW$ for N=32.

B. Additional notes

A few previsions have to be added for the thermal design section methodology :

- The thermal capacity of VO_2 was simplified by using a specific heat $C_p = 730$ J/kg/K and thermal conductivity $k_{VO_2} = 4$ W/m/K which correspond to average values over the transition. A more complicated model can be undertaken but the additional complexity wouldn't bring more insight into the thermal behavior of the antenna. The relevant point for this work is that we reach a thermal steady state after only a few microseconds. More information about thermal properties of VO_2 thin films around their transition can be found [5] .
- The thermal resistance of layer-layer interfaces is neglected, as in practice it can only increase the thermal resistance between two adjacent antennas which would help increase thermal contrast. This is a positive effect we did not take into account.
- The volumetric heat generation in the antenna is determined by the maximum current density in the top gold nanowire set to $J = 2.10^{11}\ J.m^{-2}$. We calculate Q_{vol} with the following equation $Q_{vol} = \rho.J^2$, where ρ is the electrical resistivity of gold, set to $\rho = 2,44.10^{-8}\ \Omega.m$ [6].

C. IHS model - assumptions

The details of the IHS modelling can be fund elsewhere [7][8]. Herein we describe just show here our parameters and simulation results. We also remind the reader that most of the parameters we have are engineering choices that depend on many external factors and not the outcome of a direct optimisation. We have to keep in mind that they can be adjusted if necessary to accommodate specific design requirements but this will also impact other parameters. For example, we can link the temperature contrast between adjacent antennas, the insulating layer thickness, its thermal conductivity, the heating layer conductivity and the current density during heating. If we modify one of these parameters (which is completely possible), it will affect one or more of the others.
We took a relatively conservative approach in the antenna thermal design to allow for large tolerances. For example the current density is a full order of magnitude below the experimental limit and the temperature contrast is $\Delta T =$ 25 K, a very large value that leaves plenty of room for error on either side of the transition width. We take similarly conservative specifications for the IHS design to show that our proposed design is realistic. Therefore, it is likely that the limitations of this structure investigated here can be pushed further by reducing the margins or using more advanced experimental conditions.

The IHS model calculates the thermal resistance of a heat spreader. It is put in parallel with the thermal resistance of the convection phenomenon. We therefore have to calculate the thermal resistance separately and add them to calculate the temperature increase in the array. We make the following assumptions :

- On average, half of the array's antennas will be in the ON state, the array is continuously working so that the incident heat flux is half of E_d calculated in equation 15.
- The cooling is done with air at $T_{air} = 20\ °C$, we assume $h = 10\ W/m^2/K$ which would correspond to a very light forced convection case
- The IHS model assumes heating and cooling on different sides of the heat spreader, which is not the case in our proposition. We assume the difference in thermal resistance is negligible when we have a thickness close to the optimum.
- The surface of the substrate is $A_1 = 1\ cm^2$, this is the surface of the IHS.
- The array is a square, meaning the length of the antennas increases with their number ($w_{array} = l_{array} = n.p$).
- We use gold as a substrate, which has a thermal conductivity $k_{Au} = 310$ W/m/K. Copper would actually give a lower IHS thermal resistance as $k_{Cu} = 400$ W/m/K but we conducted all the FDTD simulations using an optically thick Au backplane.

D. IHS model - calculations

The IHS model calculations include the following steps :

- Calculate dimensionless dimensions (length scales)
- Calculate dimensionless thermal resistances
- Scale up the problem with the actual dimensions
- Calculate the heat transfer equilibrium temperatures

We will follow these steps as described by [7], the derivation of the IHS model (which is an exact calculation in a 2D asymmetric geometry) is shown in [8]. The input parameters of this model are shown in Figure S7.

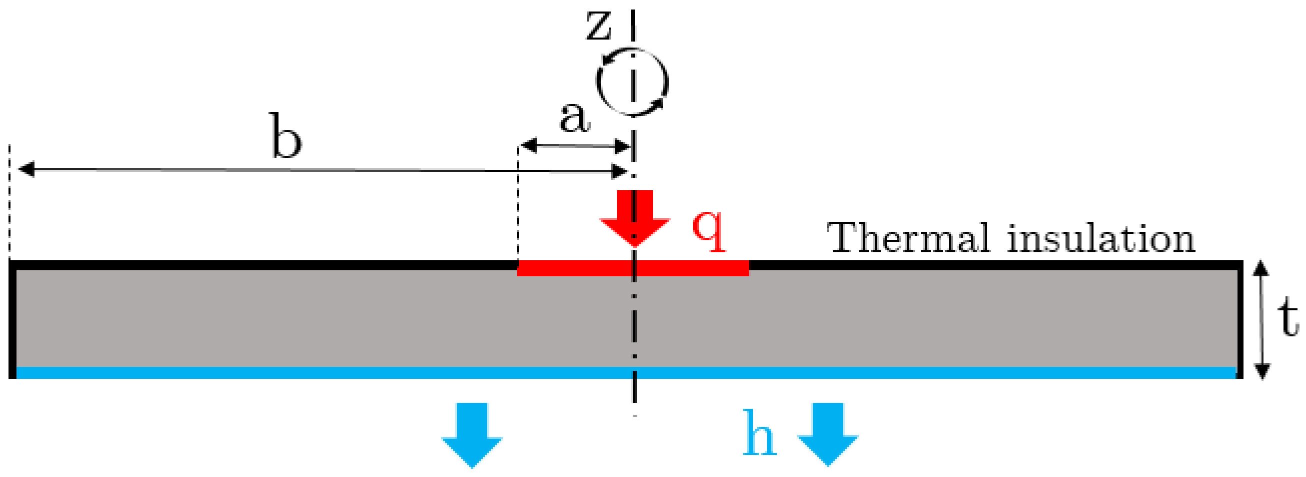

Figure S7: IHS model schematic

N	ΔT_{max}	ΔT_{avg}
16	2.3	2.3
32	8.4	8.3
64	32.1	31.9
128	125.2	124.8
256	496.0	494.9

Table S 2: Maximum and average temperature increase predicted by the IHS model for a range of number of antennas in the array

First for a non-circular source and spreader, the effective dimensions are calculated as :

$$a = \sqrt{\frac{p^2}{\pi}} \quad b = \sqrt{\frac{A_1}{\pi}} \tag{17}$$

The dimensionless heat source radius and plate thickness are respectively

$$\epsilon = \frac{a}{b} \quad \tau = \frac{t}{b} \tag{18}$$

The effective Biot number is defined as

$$Bi = \frac{h.b}{k} \tag{19}$$

From these parameters, we derive Θ and λ : two numbers used as intermediate variables that simplify down the next equations.

$$\lambda = \pi + \frac{1}{\sqrt{\pi}.\epsilon} \tag{20}$$

$$\Theta = \frac{tanh(\lambda\tau) + \frac{\lambda}{Bi}}{1 + \frac{\lambda}{Bi}.tanh(\lambda\tau)} \tag{21}$$

From these, we derive the dimensionless thermal resistances Ψ_{max} and Ψ_{avg} that are respectively used to calculate the maximum and average temperature raise over the IHS.

$$\Psi_{max} = \frac{\epsilon.\tau}{\sqrt{\pi}} + \frac{1-\epsilon}{\sqrt{\pi}}\Theta \tag{22}$$

$$\Psi_{max} = \frac{\epsilon.\tau}{\sqrt{\pi}} + \frac{(1-\epsilon)^{3/2}}{2}\Theta \tag{23}$$

We now calculate the actual thermal resistances

$$R_{max} = \frac{\Psi_{max}}{\sqrt{\pi}.x.a} \quad R_{avg} = \frac{\Psi_{avg}}{\sqrt{\pi}.x.a} \tag{24}$$

The convective heat resistance is simply

$$R_{conv} = \frac{1}{h.A_1} \tag{25}$$

Finally, we derive the thermal equilibrium temperatures :

$$T_{max} = T_{air} + q.(R_{max} + R_{conv}) \quad T_{avg} = T_{air} + q.(R_{avg} + R_{conv}) \tag{26}$$

Applying the IHS model with the parameters mentioned above, we get the following temperature increases for different array sizes shown in table 2.

This data confirms the presence of a critical array size where even the cold state has a temperature too high to be below the transition onset temperature. However, we can note that the temperature increase is governed by

convection, with our values, $R_{conv} = 1000K/W$ and R_{IHS} goes from $\simeq 200K/W$ with n=16 to $\simeq 20K/W$ with n=256. The limiting phenomenon to further cool the sample would be convection and not the IHS. This means it is easy to push for a higher number of antenna by decreasing the convection resistance, for example with higher air velocity, a larger sample size or using liquid cooling. In Figure S8 the temperature versus the distance to the IHS center is shown and it can be seen that the spread is minimal and even the edge of the IHS is at a temperature close to its center. Note that we calculated the temperature increase, which is equivalent to setting the ambient temperature (with which there is a convective heat exchange) to 0. The wires conducting electricity in the array will also dissipate heat but their impact should be small as the energy dissipation is proportional to the current density squared. As the cross section of the wires expands quickly after the array in order to be connected to macroscopic pads, the current density will decrease rapidly.

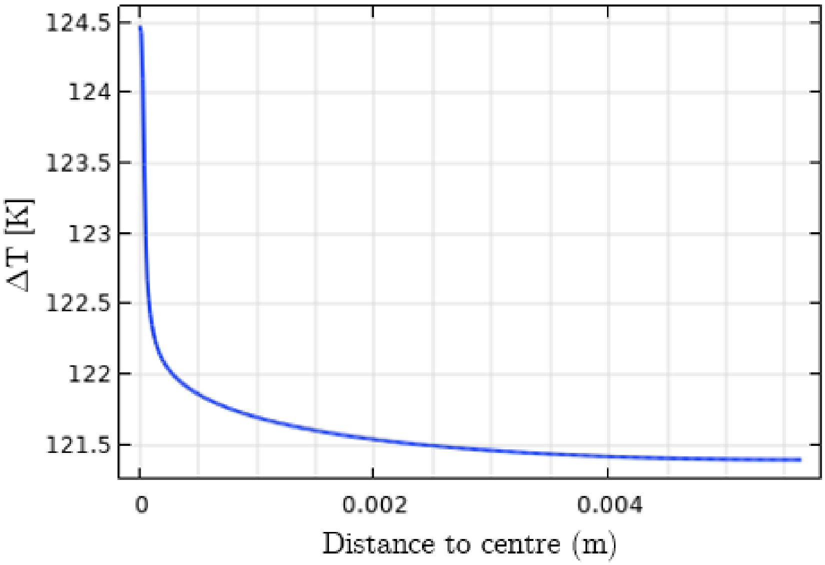

Figure S8: Temperature distribution along the IHS arc length (distance to center)

VII. Stability Analysis

To verify our design is robust to manufacturing inaccuracies, we assessed the performance of a large number of antennas built with random perturbations. This method enables us to see the cross-effects of several inaccuracies on the resonance, which individual parameter sweeps cannot do. As the number of parameters is large, we could not systematically explore nor represent this large n-dimensional space. We used a constant probability distribution for each parameter between $\pm\Delta_i$ (see Table 3) to create each individual inaccurate antenna design, the effect on the amplitude ratio and phase discrepancy are shown in Figure S9. We note that choosing a constant probability density for the manufacturing inaccuracies is a conservative approach as in reality, extreme errors are less likely to occur than smaller ones. It is however less complex and therefore more reproducible to use this method even if it overestimates the occurrence of large manufacturing inaccuracies. S

In order to further analyse this data, we can quantify the spread in phase discrepancy and reflection ratio as shown in Figure S10. A Gaussian fit applied to the phase shift discrepancy gives an average of $1.60°$ and a standard deviation of $10.5°$. Similarly for the transmission ratio we obtain an average of 0.986 and a standard deviation of 0.122. The average values are very close to the ideal ($0°$ and 1) and the standard deviations are reasonably low despite the conservative Δi chosen and the constant probability distribution that does not penalize extreme inaccuracies. From these simulations, one can be confident our design is robust to all types of fabrication inaccuracies.

Parameter	Value (nm)	Δ_i (nm)
p	516.7	20
w	245.2	20
t_{SiO2}	600	5
t_{VO2}	216.3	5
$t_{Authermal}$	60	5
$t_{Aureflector}$	99.4	5
t_{Ti}	2	1

Table S 3: Parameter values and manufacturing uncertainty used in the stability analysis

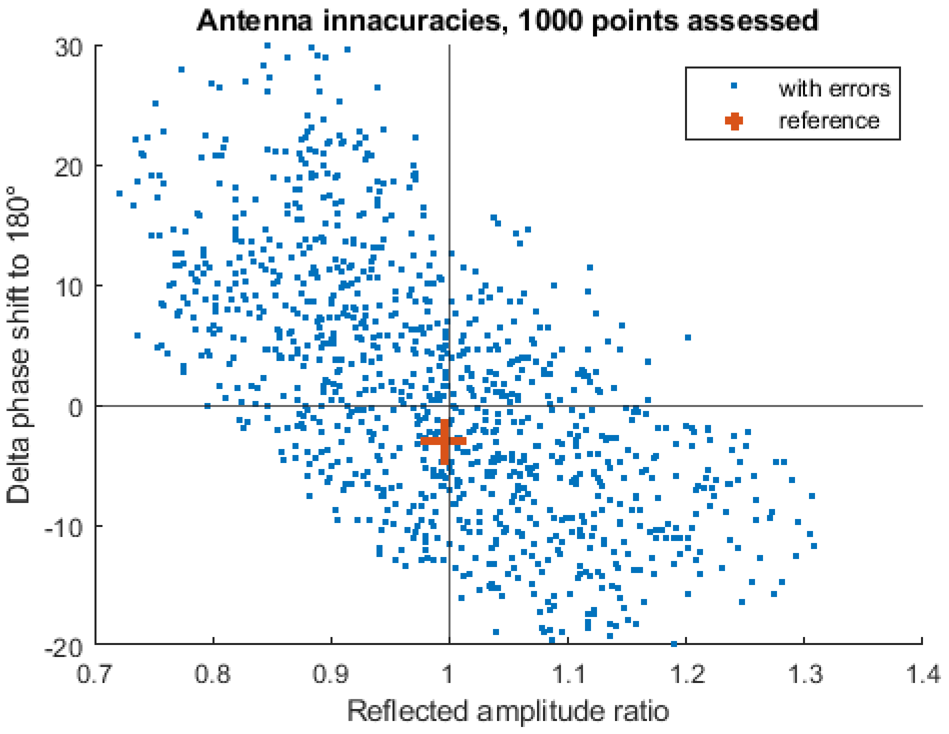

Figure S9: Stability analysis results for 1000 inaccurate antennas

VIII. Inverse design

Inverse design is a design process where the user specifies the desired performance and then uses an optimization algorithm to generate a solution. Many techniques exist in that regard, the most simple ones rely on a parametric design where some continuous features are adjusted using various optimisation algorithms. As manufacturing constraints in nanophotonics are a significant barrier, we will use this set of technique as we can ensure that a parametric design is within fabrication tolerances.

A. Algorithm choice

We can choose among several algorithms to optimise the structure, however we chose an hybrid Particle Swarm Optimisation Algorithm (PSOA) + interior point algorithm given the specificity of the problem. The former is there to search among a wide range of configurations (exploration) and the latter to fine-tune a decent design found by the PSOA (exploitation). The interior point algorithm is more computationally expensive than classic gradient descent approaches but it allows us to work in a constrained parametric space (we only used upper and lower bounds for each parameter but more complex non-linear constraints can be implemented) which is useful to

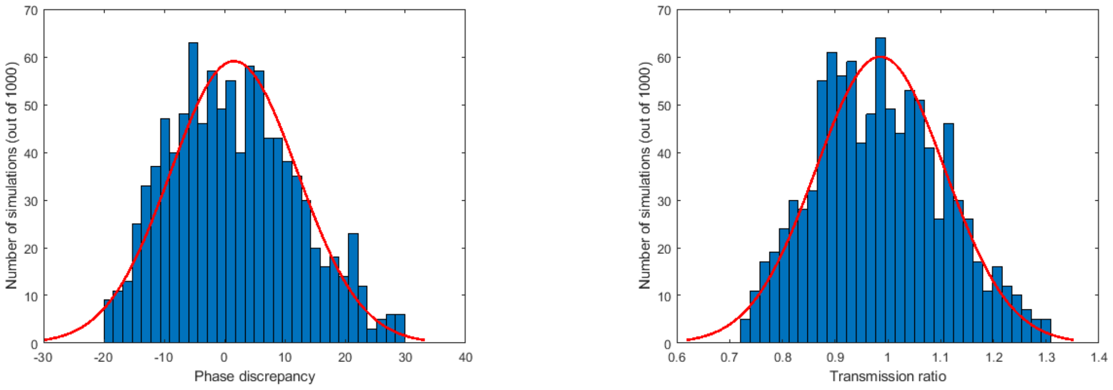

Figure S10: Phase discrepancy (left) and reflectance ratio (right) spread

respect engineering constraints.

B. Various design proposals

We varied the input parameters in the optimisation process to explore other geometries that would correspond to other engineering choices, results are shown in table 4.

- Design [2] is optimised for an incidence angle $\theta_i = 20°$, and a lower periodicity of 400 nm (like Kim et.al [9]) we observe a higher reflectance of 0.277 compared to the original design.
- Design [3] is similar to the paper proposition but at $\lambda = 905$ nm, the overall reflectance is much lower at 0.0537. Manufacturing is expected to be more difficult as the aspect ratio of the groove between adjacent antennas is much higher and the sensitivity to manufacturing imprecision is expected to scale with the design wavelength.
- Design [4] is similar to the paper proposition but the array period is now $\lambda/4$ which is 387.5 nm since the operating wavelength is 1550 nm. A higher reflectance of up to 23.9% is obtained with this shorter array period.
- Design [5] is optimised for normal incidence of $\lambda = 905$ nm, the reflectance compared to design [3] is much higher up to 12.7%.
- Design [6] has a top Au layer twice as thick as in the main design, which divides the SiO_2 thickness by 2, making it more easily manufacturable in certain cases. The reflectance drops only to 21.1%
- Design [7] is extremely similar to the original design but the material for the backplane is Copper, the change is reflectance is almost imperceptible at 0.214 instead of 0.216
- Design [8] is optimised for normal incidence at $\lambda = 1550\ nm$, the reflectance is much higher at 29.7%.

It can be inferred from these simulation results (and from other less representative attempts we did not show here for concision) that a higher reflectance can be obtained with lower array period and/or incidence angles. However, a lower array period means finer features and a higher groove aspect ratio between adjacent antennas, which is harder to manufacture, similarly, we saw that lower angles of incidence reduce the angular span of the region of interest for beam steering applications. The choice of the array periodicity and angle of incidence go beyond the optical performance indicators as manufacturing and engineering factors must be taken into account. We see that by modifying some engineering parameters, we can change the optimised thicknesses, and therefore adapt to manufacturing constraints. For example design [6] sees its SiO_2 thickness divided by two at the expense of a thicker top Au layer, VO_2 layer and Au reflector and higher energy consumption. Engineering constraints can be incorporated in the FOM and the structure performance slightly mitigated to enhance manufacturability.

Design	Original	2	3	4	5	6	7 - copper	8
θ_i	45	20	45	45	0	45	45	0
λ	1550	1550	905	1550	905	1550	1550	1550
p	$\lambda/3$	400	$\lambda/3$	$\lambda/4$	$\lambda/3$	$\lambda/3$	$\lambda/3$	$\lambda/3$
w	245.2	213.2	130.9	233.8	132.0	248.2	247.7	237.4
$t_{Au\ reflector}$	99.4	58	173.4	164.6	138.7	305.9	100.7	59.7
t_{SiO2}	600	600	600	600	600	300	600	600
t_{VO2}	216.3	124.4	133.4	124.6	85.5	195.0	217.1	170.5
$t_{Au\ heater}$	60	60	60	60	60	120	60	60
reflectance	0.216	0.277	0.0537	0.2390	0.1273	0.211	0.214	0.297

Table S 4: Alternative designs optimisation results

IX. Individual antenna wiring

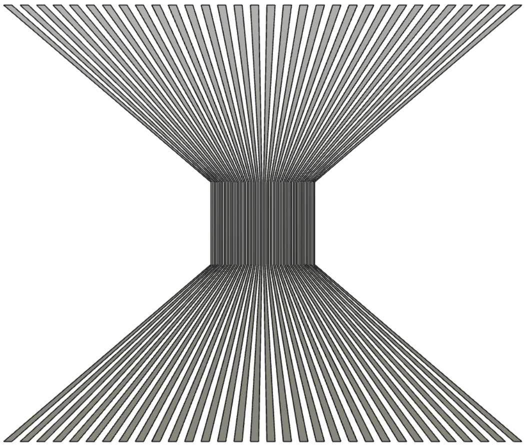

Figure S11: Array wiring proposition for N=32

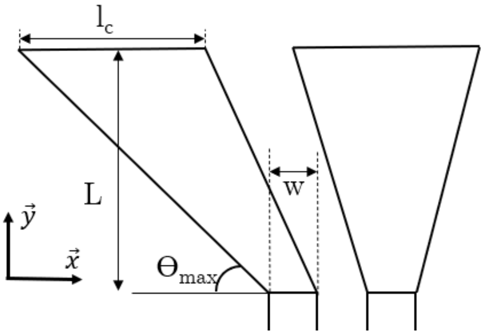

Figure S12: Array wiring schematic

In this section, we show how the antennas are wired up individually and briefly calculate the impact on the energy consumption and heat generation. The wires should expand from the array to the outside and have an increasing width to allow contact pads to be installed and to reduce the current density and therefore the energy consumption as shown in figure S11. We show a simple wire generation algorithm (that can probably be optimised in future work) and calculate the energy losses in the wiring system.

The wires are modeled as trapezoids, that are characterised by θ_{max}, L and w as defined in Figure S12. We assume $L \gg w$ and that the maximum wire width is equal to the spacing between them : the wire width varies along y uniformly between w and $L.cos(\theta)/N$, we multiply by the Au heater to find the cross section. The ith wire has approximately a length $L_i = \sqrt{L^2 + (2 * |N/2 - i + 0.5| * L/N/tan(\theta_{max}))^2}$. From this simplistic model, we can calculate the resistive losses Q_i in the ith wire by integrating them over the length of the wire.

$$Q_i = \int_0^{L_i} = \frac{\rho.I_0^2}{S(l)}dl \tag{27}$$

Where $S(l) = \dfrac{L.cos(\theta)}{N.L_i}.l$, the contact length is calculated below :

$$l_c = \frac{L}{N.tan(\theta_{max})} \tag{28}$$

The wires have to be long enough to achieve a contact length above a certain threshold, but no longer than required as any extra length will increase the resistive power losses. A small θ_{max} is required to enlarge the wires as rapidly

as possible to increase their cross section and reduce the resistive losses but small θ_{max} values also yield longer wires which increase the losses. We calculate the contact length and power losses for a range of θ_{max} and L, this brute force approach is rather quick given the simplicity of the calculations. If we choose a contact length $l_c = 100\ \mu m$, the θ_{max}-L combination that minimises the resistive losses down to 32mW in total (when half the antennas are ON) is $L = 1848\ \mu m$ and $\theta_{max} = 30°$ for N=32. This compares to an energy consumption in the array of only 3.8 mW, however the energy density in the array is extremely high (1390 W/cm^2) compared to the wiring system, spread over a much larger surface area (0.131 W/cm^2): this is why we ignore the heat generation in the contact wires in the IHS calculations. The value of $\theta_{max} = 30°$ seems to be optimal for all array sizes with our direct calculation approach.

References

[1] Hooman Abediasl and Hossein Hashemi. Monolithic optical phased-array transceiver in a standard SOI CMOS process. *Optics Express*, 23(5):6509–6519, March 2015. Publisher: Optica Publishing Group.

[2] Farshid Ashtiani and Firooz Aflatouni. N × N optical phased array with 2N phase shifters. *Optics Express*, 27(19):27183–27190, September 2019. Publisher: Optica Publishing Group.

[3] Steven A. Miller, You-Chia Chang, Christopher T. Phare, Min Chul Shin, Moshe Zadka, Samantha P. Roberts, Brian Stern, Xingchen Ji, Aseema Mohanty, Oscar A. Jimenez Gordillo, Utsav D. Dave, and Michal Lipson. Large-scale optical phased array using a low-power multi-pass silicon photonic platform. *Optica*, 7(1):3–6, January 2020. Publisher: Optica Publishing Group.

[4] Taehwan Kim, Pavan Bhargava, Christopher V. Poulton, Jelena Notaros, Ami Yaacobi, Erman Timurdogan, Christopher Baiocco, Nicholas Fahrenkopf, Seth Kruger, Tat Ngai, Yukta Timalsina, Michael R. Watts, and Vladimir Stojanović. A Single-Chip Optical Phased Array in a Wafer-Scale Silicon Photonics/CMOS 3D-Integration Platform. *IEEE Journal of Solid-State Circuits*, 54(11):3061–3074, November 2019. Conference Name: IEEE Journal of Solid-State Circuits.

[5] Dong-Wook Oh, Changhyun Ko, Shriram Ramanathan, and David G. Cahill. Thermal conductivity and dynamic heat capacity across the metal-insulator transition in thin film VO2. *Applied Physics Letters*, 96(15):151906, April 2010.

[6] Physics (4th Edition, Volume 1) by John D. Cutnell; Kenneth W. Johnson: Good Paperback (1997) | Ergodebooks.

[7] A. J. Robinson, J. Colenbrander, R. Kempers, and R. Chen. Solid and Vapor Chamber Integrated Heat Spreaders: Which to Choose and Why. *IEEE Transactions on Components, Packaging and Manufacturing Technology*, 8(9):1581–1592, September 2018.

[8] Clemens J. M. Lasance. How to Estimate Heat Spreading Effects in Practice. *Journal of Electronic Packaging*, 132(3), September 2010.

[9] Yonghwi Kim, Pin Chieh Wu, Ruzan Sokhoyan, Kelly Mauser, Rebecca Glaudell, Ghazaleh Kafaie Shirmanesh, and Harry A. Atwater. Phase Modulation with Electrically Tunable Vanadium Dioxide Phase-Change Metasurfaces. *Nano Letters*, 19(6):3961–3968, June 2019.
